# Tissue recovery practices and bioburden: a systematic review

**DOI:** 10.1007/s10561-016-9590-5

**Published:** 2016-10-19

**Authors:** S. Brubaker, K. Lotherington, Jie Zhao, B. Hamilton, G. Rockl, A. Duong, A. Garibaldi, N. Simunovic, D. Alsop, D. Dao, R. Bessemer, O. R. Ayeni, on behalf of the Bioburden Steering Committee and Tissue Recovery Working Group, Amber Appleby, Scott Brubaker, Jeannie Callum, Graeme Dowling, Ted Eastlund, Margaret Fearon, Marc Germain, Cynthia Johnston, Ken Lotherington, Ken McTaggart, Jim Mohr, Jutta Preiksaitis, Michael Strong, Martell Winters, Kimberly Young, Jie Zhao, Scott Brubaker, Ken Lotherington, Jie Zhao, Gary Rockl, Brian Hamilton

**Affiliations:** 1 American Association of Tissue Banks, 8200 Greensboro Drive, Suite 320, Mclean, VA 22102 USA; 2 Canadian Blood Services, 270 John Savage Ave., Dartmouth, NS B3B 0H7 Canada; 3 Comprehensive Tissue Centre, 11402 University Avenue, Edmonton, AB T6G 2J3 Canada; 4 Musculoskeletal Transplant Foundation, 125 May St., Suite 300, Edison, NJ 08837 USA; 5 Southern Alberta Tissue Program, Foothills Medical Center, McCaig Tower Rm 4410, 1403 29th St. NW, Calgary, AB T2N 2T9 Canada; 6 Department of Surgery, McMaster University, 293 Wellington St. N, Suite 110, Hamilton, ON L8L 8E7 Canada; 7 McMaster University Medical Centre, 1200 Main St W, Room 4E15, Hamilton, ON L8N 3Z5 Canada

**Keywords:** Allograft, Tissue recovery, Tissue processing, Allograft contamination

## Abstract

For successful transplantation, allografts should be free of microorganisms that may cause harm to the allograft recipient. Before or during recovery and subsequent processing, tissues can become contaminated. Effective tissue recovery methods, such as minimizing recovery times (<24 h after death) and the number of experienced personnel performing recovery, are examples of factors that can affect the rate of tissue contamination at recovery. Additional factors, such as minimizing the time after asystole to recovery and the total time it takes to perform recovery, the type of recovery site, the efficacy of the skin prep performed immediately prior to recovery of tissue, and certain technical recovery procedures may also result in control of the rate of contamination. Due to the heterogeneity of reported recovery practices and experiences, it cannot be concluded if the use of other barriers and/or hygienic precautions to avoid contamination have had an effect on bioburden detected after tissue recovery. Qualified studies are lacking which indicates a need exists for evidence-based data to support methods that reduce or control bioburden.

**Electronic supplementary material:**

The online version of this article (doi:10.1007/s10561-016-9590-5) contains supplementary material, which is available to authorized users.

## Introduction

There are 13 Canadian tissue banks registered with Health Canada that recover human tissue from donors for use in transplantation, and 8 of them are accredited by the American Association of Tissue Banks (AATB). Commonly recovered tissues include skin, cardiac (the heart), bone, and fibrous connective tissue. The purpose of Health Canada’s regulatory initiative involving human tissues is to minimize the potential health risks to Canadian recipients of tissue allografts. The regulations establish safety requirements such as tissue retrieval (recovery) practices, among other critical steps, to improve the “protection of the health and safety of Canadian transplant recipients” (Government of Canada 2013). The complete microorganism profile of an allograft is termed the tissue’s bioburden. While bioburden represents the type and quantity of microorganisms associated with an allograft sample, contamination denotes the simple presence of microorganisms on or in the sample. Reduction due to antimicrobial intervention (i.e., disinfection) can be assessed qualitatively in relation to improvement in contamination rate, or quantitatively by determining the bioburden load before and after an intervention. Tissues removed under aseptic conditions may still be determined to be contaminated following recovery and/or processing, which contributes to allografts determined to be unsuitable (Mroz et al. 2008). To maximize safety and prevent the loss of tissue allografts, tissue banking professionals have implemented procedures and disinfection methods to control, reduce or eliminate bioburden and, where possible, have established minimum sterility assurance levels for sterilization methods with a focus to reduce the likelihood of provision of a contaminated allograft for transplantation.

There are a variety of approaches available to test tissue and determine allograft bioburden. The “Guidance Document for Cell, Tissue and Organ Establishments—Safety of Human Cells, Tissues and Organs for Transplantation” published by Health Canada (2013) describes responsibilities for registration, donor screening and testing, processing, packaging, labeling, quarantine, storage, reporting, records, and quality assurance systems. In the Canadian regulations, tissue recovery is included within the broad scope of “processing.” Initially, donor eligibility is assessed and includes screening for infectious disease risks based on medical and social (behavioral) history, physical examination, the results of any diagnostic procedures performed, and, if applicable, an autopsy (Government of Canada 2013). The guidelines recommend an interview and questionnaire that includes screening for risks associated with contraindications/exclusion criteria. In addition, donors must be tested for communicable diseases/infections including human immunodeficiency virus-1 and -2, hepatitis B and C viruses, and other communicable diseases, using commercial tests that are appropriate and effective. Upon recovery of certain tissue types from a donor, it’s customary to assess microbiological contamination by obtaining a swab culture of each tissue, and the swab is later inoculated to a culture medium to promote growth and determine bioburden. Microbiological culture results can guide interventions to minimize the risk of a bacteriological pathogen transmission to a recipient. Additional tests may be performed to evaluate or provide information prior to processing or storage in a tissue bank. Relative to Canadian tissue banks, recovered tissues (such as a heart, bone, fibrous connective tissue or skin) can be fashioned into multiple allografts for transplantation (e.g. the dissection of a heart into one to four cardiac allografts for transplantation into as many recipients; bone can be cut and sized for use in more than a dozen recipients).

Although allografts have been extensively used for transplantation worldwide, best practices for tissue recovery to control or prevent contamination and cross-contamination have not been thoroughly examined and published. One study showed a trend that an increased number of people in the operating room during recovery increased musculoskeletal allograft contamination, suggesting that recovery variables may impact contamination rates and bioburden (Segur et al. 2000). In the Canadian guidelines, control of contamination is expected, but specific methods are not described.

In this review, we examined the methods used to recover tissue from donors in an effort to determine the most appropriate procedures that could minimize allograft contamination/bioburden and optimize transplantation potential.

## Methods

### Information sources and search

The search strategy was developed and reviewed by SF and the tissue recovery working group at Canadian Blood Services. The search was applied to the electronic databases MEDLINE and EMBASE from 1974 to July 25, 2014 including the following search terms: “tissue recovery,” “bioburden reduction,” “bioburden control,” “aseptic technique,” “cleaning,” “tissue banking,” “storage,” and “asystole”, among others. An additional reviewer (AG) performed an updated search using the original search strategy to include publications up to March 6, 2015. The full search strategy is shown in Appendix A.

### Study selection

Eight reviewers (KL, SM, SB, GR, BH, SF, CH and JZ) independently screened each of the citations in duplicate to identify studies that met all of the following four inclusion criteria: (1) evaluated bone, fibrous connective tissue, cardiovascular tissue or skin; (2) included a tissue recovery parameter such as ischemic time, body cooling, donor condition, skin preparation, recovery site, instruments, and/or attire of recovery personnel; (3) included a storage or transport parameter such as storage medium or temperature, packaging, shipping container or procedure, refrigerant to tissue ratio, duration of storage and/or transportation; and (4) included outcomes in either patient infection attributed to tissues, proportion of allograft that did not meet release criteria due to bioburden load, or microbe detection on tissues (bioburden rates, log reduction, counts, antibiotic potency, initial contamination rate versus final). A study was excluded if it was an editorial, letter, conference abstract, or review. During duplicate screening, if there was disagreement, the full report was retrieved and an independent assessment was repeated until consensus was reached.

### Data abstraction

Design of data abstraction forms and evidence tables were guided by the questions in the analytic framework (Appendix B). Three reviewers (RB, DA and AD) independently collected study characteristics including the donor type, recovery site, tissues collected, cold ischemic time, and warm ischemic time. The bioburden analysis was summarized for each study and data included sample preparation and method, incubation period and temperature, species identified, bioburden, and proportion of samples not suitable for transplantation. Any discrepancies in data abstraction were resolved by consensus.

### Quality assessment

Following the screening process, clinical studies that met the eligibility criteria were evaluated for quality using the Grades of Recommendation, Assessment, Development, and Evaluation (GRADE) assessment. The GRADE quality of a study is dependent on the consistency of the results, directness of the evidence, and precision to inform recommendations (Guyatt et al. 2011). There is no validated quality assessment tool for laboratory-based studies because basic science research is inherently considered level IV, or low quality evidence (Balshem et al. 2011).

### Data analysis

Data abstracted from studies that qualified were organized into tables presenting study characteristics, bioburden analysis, recovery, storage and transport, and finally, outcomes. Descriptive statistics include the frequency and percentage of bioburden outcomes, as well as mean proportions. A meta-analysis was not performed due to high heterogeneity among clinical studies.

## Results

### Study selection

**Fig. 1:**
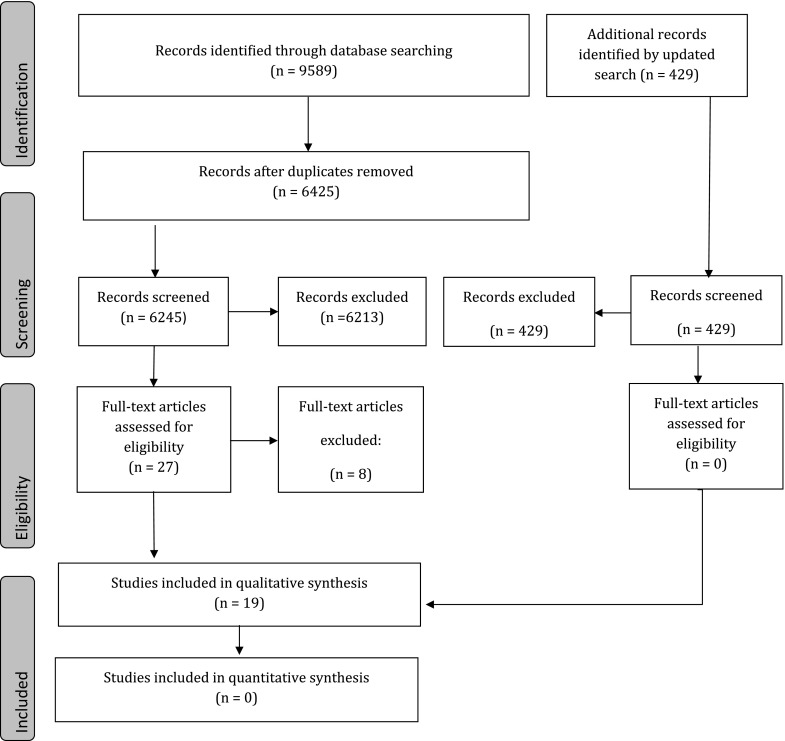
Summary of search strategy

A total of 6245 citations were reviewed after duplicates were removed (Fig. 1). Twenty-eight citations were selected for full text review, of which 19 were included. These include seven laboratory studies and 12 clinical studies all of which are elaborated below. Of the 28 citations, nine were excluded because they did not fulfill the screening criteria and are described in Appendix C. Following the updated search to include articles up to March 6, 2015, an additional 429 articles were retrieved; however, none were identified for full text evaluation.

### Characteristics and culture methods of the studies

The studies selected were published between 1992 and 2013. Almost half of them (9/19) were from Europe, with the remaining from Australia (4/19), Asia (3/19) and North America (3/19). Of the 19 studies, seven were laboratory studies and 12 were clinical studies (Tables 1 and 2).

**Table 1:** Characteristics of laboratory studies

First author, year	Country	Sites	Donor	Location of recovery	Tissue(s) recovered
Gaucher et al. (2012)	France	1	Multi-organ	NR	Skin
Jashari et al. (2007)	Belgium	2	Cadaveric living	Morgue operating theatre	Heart, artery
Castagnoli, Castagnoli et al. (2003)	Italy	1	Multi-organ	NR	Skin
Bravo et al. (2000)	USA	1	Cadaveric	NR	Skin
Wester et al. (1998)	USA	1	Cadaveric	NR	Skin
Armiger (1995)	New Zealand	1	Cadaveric	NR	Cardiac (pulmonary and aortic valves)
Niwaya et al. (1995)	Japan	1	Organ	NR	Cardiac (pulmonary valves)

*NR* Data not reported

**Table 2:** Characteristics of clinical studies

First author, year	Country	Sites	Clinical study type	Level of evidence	Donor	Recovery site(s)	Tissue(s) recovered
Chapman and Villar (1992)	England	1	Cohort	II	Cadaveric living	NR	Bone (femoral heads, massive allografts; during hip replacement or general organ harvest)
Heng et al. (2013)	Singapore	1	Retrospective	III	Organ living	OR or designated clean room	Heart
Schubert et al. (2012)	Belgium	1	Retrospective	III	Multi-organ	OR or tissue recovery room	Bone and tendon (following organs)
Gocke (2005)	USA	1	Retrospective	III	Organ	OR	Bone, tendons, fascia, others
Goffin et al. (2000)	Belgium	1	Retrospective	III	Cadaveric, multi-organ living	NR	Heart
Sommerville et al. (2000)	Australia	1	Retrospective	III	Living	OR	Bone
Journeaux et al. (1999)	UK	1	Retrospective	III	Cadaveric, Multi-organ Living	NR	Bone
Verghese et al. (1998)	India	1	Retrospective	III	Cadaveric living	Mortuary	Cardiac
Bettin et al. (1998)	Germany	1	Retrospective	III	Multi-organ cadaveric	OR, morgue	Bone (after explantation of parenchymatous tissue)
Goffin et al. (1996)	Belgium	NR	Retrospective	III	Cadaveric multi-organ living	NR	Heart
Campbell and Oakeshott (1995)	Australia	1	Retrospective	III	Cadaveric living	OR, autopsy room	Bone
Gall et al. (1995)	Australia	1	Retrospective	III	Cadaveric multi-organ living	OR, mortuary/clean room	Heart (prior to postmortem evaluation)

Quality of evidence, Level I evidence: high, Level II evidence: moderate, Level III evidence: low, Level IV evidence: very low

*OR* operating room, *NR* not reported

This review assessed the recovery methods for multiple tissue types, which included heart (746 laboratory samples and 3857 samples for transplantation), bone (zero laboratory samples and 303,551 samples for transplantation), skin (448 laboratory samples and zero samples for transplantation) and, finally, fibrous connective tissue (zero laboratory samples and 3315 samples for transplantation). Skin allografts were included in four of the 19 studies, whereas eight addressed cardiac tissues, and seven studies analyzed musculoskeletal tissues.

The site where tissue recovery took place varied. Recovery was performed in operating theatres for living and organ donors, and in a morgue or an operating room for cadaveric donors.

In all 19 studies, the presence of microorganisms (including bacteria, and fungi) was determined by placing pieces of tissue directly in culture media, or by swabbing the tissue and placing the swab in culture media. In one laboratory study, tissue fragments were cultured to determine bioburden following recovery (Jashari et al. 2007). Culturing details were not reported in the remaining six laboratory studies. In the 12 clinical studies, two studies cultured tissue samples directly, one study cultured tissue swabs, three studies cultured tissue samples and swabs, two studies cultured tissues and tissue rinses or solutions, and four studies did not report the sampling method for culturing.

There was a variety of growth media used to culture microorganisms (Appendix D). One laboratory study reported thioglycollate media to culture bacteria, and Sabouraud, trypto-casein and soy media for culturing of fungi and yeast (Jashari et al. 2007). For clinical studies, media types included thioglycollate agar, Sabouraud agar, blood agar, chocolate agar, brewer’s liquid culture media, reinforced clostridial media, Stuart’s transport media, dextrose broth, Schaedler’s broth, Kimmig plates or endo agar. Incubation of cultures at temperatures between 20 and 37 °C for one to 21 days was reported in nine studies. Six laboratory studies and four clinical studies did not report incubation parameters.

### Study outcomes

#### Contamination rates, bioburden and pre-recovery conditions

Contamination rate following tissue recovery was reported in only one laboratory study (Appendix E1). This study found contamination rates in the transport media following heart tissue recovery was an average of 26.7 % (Jashari et al. 2007). The rates of contamination following recovery were reported in 12 clinical studies (Appendix E2). Cardiac tissue contamination rates were on average, 19.9 % (range 13.9–42.9 %). In addition, 54 % of heart valves recovered in a morgue from multi-organ donors were reported as contaminated, while those in an operating room were contaminated at a rate of only 12 % (Gall et al. 1995). Samples of recovered bone allografts were contaminated an average of 19.4 % (Std. dev: 15 %; range 4.7–49 %). One study examining fibrous connective tissues (tendons) observed a contamination rate of 2.7 % (Schubert et al. 2012).

The most commonly reported genera of microorganisms identified in 10 clinical studies and one laboratory study were (in order of prevalence): *Staphylococci, Streptococci, Pseudomonas*, *Bacillus*, *Candida*, *Acinetobacter*, *Escherichia*, *Propionibacterium*, *Enterococci* and *Corynebacterium*. Across studies there was no apparent relationship between species of microorganisms and pre-recovery conditions. However, one study observed a higher rate of pathogenic microorganisms (e.g. *Staphylococcus aureus, Escherichia coli*, *Candida,* etc.) associated with allografts recovered in the morgue compared to recovery that took place in an operating room (33 and 20 %, respectively), and conversely a higher rate of non-pathogenic microorganisms (e.g. *Propionibacterium, Corynebacterium,* etc.) in allografts recovered in the operating room compared to recovery in the morgue (31 and 7 %, respectively) (Bettin et al. 1998).

Contamination rates may be correlated with the site of tissue recovery. One study observed lower contamination rates in heart valves recovered from multi-organ donors in operating rooms (12 %) compared to valves recovered from cadavers in an open mortuary (54 %) (Gall et al. 1995). Another study found that cardiac tissue recovered in the operating room from multi-organ donors had contamination rates of 21.7 %, while tissue recovered from non-heart beating (cadaveric) donors in the morgue had a higher contamination rate of 33.3 % (Jashari et al. 2007). In addition, studies with the lowest proportions of contaminated tissues following recovery (2.7, 4.7 and 5 %) were performed in operating rooms (Gocke 2005; Schubert et al. 2012). Conversely, Bettin et al. 1998 reported that tissues recovered in operating rooms were more contaminated (51 %) than tissues recovered in morgues (40 %). However, as described above, the study also showed that microorganisms contaminating allografts from the morgue were more likely to be pathogenic compared to the operating room. Other confounding factors (e.g., trauma) that could affect contamination rates were not analyzed.

Details regarding warm ischemic time (time period from asystole to subjecting the tissue to cold rinse or transport) were reported for five laboratory studies and seven clinical studies. In clinical studies, the warm ischemic time was consistently less than 24 h. Recovery of skin tissue was performed within 24 h following death in laboratory studies, and the warm ischemic time was less than 15 h. Warm ischemic time periods for bone tissue were less than 24 h. Similarly, connective tissue was recovered within 24 h of death in clinical studies (Schubert et al. 2012).

Cold ischemic time (the time interval from subjecting cardiac tissue to cold rinse or transport solution at recovery to the beginning of disinfection) was more varied than that of the reported warm ischemic times. The cold ischemic time period for skin tissue ranged 0.5–24 h, and was less than 24 h for cardiac tissues in laboratory studies (Bravo et al. 2000; Castagnoli et al. 2003; Gaucher et al. 2012; Jashari et al. 2007).

Other procedures utilized to minimize tissue contamination did not appear to correlate with contamination rates, however, the details provided for each study varied. For example, one laboratory study indicated that recovery was performed with sterilized equipment, although other precautions against contamination were not reported, and resulted in an average contamination rate of 26.7 % (Jashari et al. 2007). Others such as Bettin et al. (1998) reported a number of precautions including the use of two sets of instruments, double-gloves, skin decontamination and sterile drapes. Despite these precautions, researchers still observed relatively high allograft contamination rates of 40 and 51 % of samples (cadaveric and organ donors, respectively) (Bettin et al. 1998). Other methods to reduce contamination included ensuring that the sternotomy did not enter the trachea or abdominal cavity during cardiac tissue recovery, or the use of sterilized gloves, gowns, or draping (Gall et al. 1995; Heng et al. 2013; Verghese et al. 1998).

Two studies indicated decontamination of the skin prior to tissue recovery. One study applied a 10 % povidone iodine solution to the skin for 15 min while the other simply indicated that the skin was “sterilized” for post-mortem tissue recoveries (Bettin et al. 1998; Gall et al. 1995). No other studies reported pre-recovery skin decontamination procedures to minimize contamination.

#### Tissue recovery/pre-processing storage and contamination rates

Following recovery, tissues were stored in a variety of different solutions prior to processing. Recovered cardiac tissues were immediately submerged in fluid within sterile plastic bags in two studies (appendices F and G) (Jashari et al. 2007; Verghese et al. 1998). Two studies reported the use of saline, Ringer’s solution, Eurocollins, UW, or tissue culture medium 199 with HEPES buffer for storage (Goffin et al. 1996, 2000). Jashari and associates utilized similar isotonic storage solutions like saline, Ringer’s solution, tissue culture medium 199, and Hank’s balanced salt solution, but also indicated that solutions were constantly “maintained at 4 °C” using ice.

Similar to cardiovascular tissue, tissue banks opted to maintain the storage temperature of skin at 4 °C, when reported (Bravo et al. 2000; Castagnoli et al. 2003; Gaucher et al. 2012; Wester et al. 1998). The recovered skin was submerged in Eagle’s minimum essential medium (MEM) with Earle’s balanced salt solution (BSS) without antibiotics at 4 °C following recovery in only one study (Wester et al. 1998). In the remainder of the studies evaluating skin samples, recovered tissue was submerged in solutions containing antibiotics. Rinsing of tissue (with saline) following recovery was only reported by one study. The rinsed samples were then submerged in X-Vivo tissue culture medium with gentamicin (80 µg/ml) and vancomycin (500 µg/ml) (Bravo et al. 2000). In the remainder of studies, recovered skin was placed into RPM1640 medium with 1 % serum albumin (supplemented with 100 µg/ml vancomycin, 50 µg/ml trimethoprim-sulfametoxazole, 50 µg/ml fluconazole) or Ringer’s lactate solution or RPMI 1640 medium supplemented with gentamicin (320 mg/l), vancomycin (500 mg/l) and lincomycin (600 mg/l) (Castagnoli et al. 2003; Gaucher et al. 2012).

The transport and storage parameters of recovered bone tissue were only reported in one study. Recovered bone tissue was triple-wrapped in plastic bags, containers, or both and stored at −70° (Campbell and Oakeshott 1995).

### Quality of clinical studies

Of the 12 clinical reports, one performed a prospective cohort analysis (level II evidence) while the remaining studies were retrospective analyses (level III evidence). Using the GRADE assessment, the quality of clinical studies according to the objectives was found to range from very low to moderate (Table 2). The clinical studies that addressed the time period between asystole to skin preparation for tissue recovery were of very low, to low quality. Additionally, clinical studies that investigated the effect of body cooling or warm ischemic times were of low quality. Clinical studies that addressed skin preparation parameters, or that included use of barriers to reduce contamination, provided very low to moderate quality evidence. The clinical studies that addressed excision techniques, and post recovery storage conditions were of low quality.

### Confounding effects

Within an individual study many procedures are standardized, but bioburden is rarely evaluated prior to, and immediately following each recovery and processing step. This makes it difficult to determine their impact on bioburden. Of the 19 studies in this review, bioburden following recovery or following any bioburden reduction process was not reported. Often, only the contamination rate was reported, which provides insight into the number of samples that are contaminated, but does not address the efficacy of any methods to reduce the proportion of contaminants, such as pre-recovery skin preparation, or even warm ischemic time. Therefore, the efficiency of decontamination efforts within the study cannot be determined.

Similarly, the inclusion of multiple methods related to one outcome also makes it difficult to assess the efficacy of each method used to reduce bioburden. Warm ischemic time, contamination barriers, skin decontamination methods, skin recovery personnel precautions, and recovery methods were all assessed for their contribution towards the bioburden following recovery. In each study, a standard protocol was chosen for each of these parameters. The lack of a reference or control method greatly inhibits the confidence with which a process can be recommended. Among different studies, the lack of consistency in recovery protocols makes it difficult to determine the most important variable(s) during the tissue recovery process.

Additionally, the type of tissue recovered, as well as the environment that the tissues were recovered in, could affect bioburden outcome. Although most studies reported isolation of tissues using aseptic techniques, isolation of different tissues require different protocols, and include different potential sources of contamination.

Finally, variables such as donor skin condition (abrasions, lacerations), presence of medical interventions, cleanliness of skin, trauma, compound fractures, and how they might correlate to bioburden load were not reported.

## Discussion

In this review, methods to reduce bioburden and the contamination rate prior to, during, and following tissue recovery were reviewed. The lowest rate of contamination was found when warm ischemic time was maintained below 24 h, and a team of two to six surgeons performed the recovery of the bone tissue. In one case report, the contamination rate was lowest when five recovery personnel were involved in the recovery process. Additional precautions, such as maintaining warm ischemic times below 15 h, the use of operating rooms as recovery sites, taking extra care to not enter the abdominal cavity of the donor during recovery of heart valves, or the use of sterile draping during recovery, also demonstrated a lower contamination rate (13.9 %). Interestingly, one of the highest rates of contamination (average 46.7 %) was observed when recovery precautions included the use of two sets of instruments for recovery, double gloves, sterile draping, shaving and scrubbing procedures, and the use of 10 % povidone iodine for 15 min to decontaminate the skin, despite maintaining a recovery time less than 24 h post asystole, and a maximum warm ischemic time of 12 h.

Contamination rates may be correlated with the site of tissue recovery. One of the studies observed lower contamination rates in heart valves recovered from multi-organ donors in operating rooms (12 %) compared to valves recovered from cadavers in an open mortuary (54 %) (Gall et al. 1995). Reduced contamination during recovery in operating rooms was also observed in three other studies (Gocke 2005; Jashari et al. 2007; Schubert et al. 2012). Some studies attribute this difference not to the room itself, but instead to the classification of the donor (i.e., tissue recovery from a multi-organ donor or living donor occurs in an operating room, whereas a deceased donor of tissues may be located in a morgue when recovery takes place) (Journeaux et al. 1999; Schubert et al. 2012). In contrast, Gall et al. (1995) attributes the increased contamination in the morgue to the atmosphere of the room, given that the most common species identified were skin and respiratory flora (Gall et al. 1995). One study reported the opposite trend of higher contamination in the operating room compared to the morgue (51 % and 40 %, respectively) (Bettin et al. 1998). However, the study also showed that microorganisms contaminating allografts from the morgue were more likely to be pathogenic (e.g. *S. aureus, E. coli, Candida*) compared to the operating room (Bettin et al. 1998). Within North America, the American Academy of Orthopaedic Surgeons states that tissue should be recovered aseptically in an operating room and not the morgue, and within 24 h of the donor’s death (Gitelis and Wilkins 2011). The reports in this review support these standardized practices, despite potentially higher contamination rates with non-pathogenic microorganisms.

One variable of tissue recovery that may impact contamination is time from asystole to time of tissue recovery and whether body cooling occurred. Currently, the literature is not sufficient to determine whether the time interval from asystole to tissue recovery or warm ischemic time is a better predictor of contamination; however, studies have indicated that in general, a reduction in the time period between asystole to tissue recovery is an important contributor to contamination. For example, heart valves recovered from non-heart beating donors (<6 h warm ischemic time, or <36 h recovery time) had a significantly lower contamination rate compared to valves from beating-heart donors (i.e. the interval from asystole to tissue recovery is considered to be near to 0 h) (Goffin et al. 2000). Another study recommended bone recovery within 24 h of asystole to reduce the overgrowth of skin microorganisms (Journeaux et al. 1999). Vehmeyer et al. (2002) have demonstrated that the risk of blood contamination increased each hour following asystole (cessation of heart beating), suggesting post-mortem time to recovery should be kept to a minimum (Vehmeyer et al. 2002). Similarly, the warm ischemic time has been kept to a minimum in all studies, when reported, as some studies have shown that cooling the body may reduce the bioburden. At lower temperatures, the growth rate of many bacteria is diminished (Ratkowsky et al. 1983, 1982).

Other pre-recovery variables did not appear to be correlated with a decrease in contamination rates. For example, the use of skin decontamination steps or physical barriers did not appear to decrease contamination rates. Higher rates of contamination were reported when skin decontamination was performed (average contamination rate of 52.5 %) compared to other studies where a method to decontaminate skin was not reported (average contamination rate of 19.9 %).

Different tissues may experience different rates of contamination. The average contamination rates of cardiac tissues are slightly greater than bone (approximately 28 vs. 23 %), but this may be attributed to a more intensive culture protocol (Sommerville et al. 2000). The highest rates of bone contamination occurred in allografts that were recovered after the removal of organs (Bettin et al. 1998). Contrarily, Segur et al. (1998) observed that the initial removal of other organs did not impact bone contamination, suggesting other factors may be important for contamination during recovery.

### Limitations

In this systematic review, the most effective methods of tissue recovery to minimize contamination are addressed. Overall, the quality of included studies ranged from low to moderate. No data regarding tissue recovery order, donor skin condition, presence of trauma or compound fractures, or the hygiene, presence of an acute illness, or open lesions in tissue recovery personnel were reported.

The contamination rate outcome was utilized to determine the most effective methods to reduce contamination following recovery. There was a large amount of heterogeneity in the culturing methods to identify the presence of microorganisms. Some organisms are extremely fastidious, and may only grow within a narrow range of nutrient and environmental conditions. Most studies used media types that are proposed to be able to capture the majority of organisms that may contaminate the tissues, but the use of only one culture medium or incubation parameter could possibly exclude important pathogens that would affect transplantation outcomes. The swab culture method has been reported to have a low recovery efficiency (9–10 %), suggesting that it is unreliable for the detection of the majority of microorganisms (Veen et al. 1994). Additionally, some organisms are extremely fastidious, and may only grow within a narrow range of nutrient and environmental conditions, which can further reduce detection. Sommerville et al. (2000) noted that the development of deep infections in allograft recipients were due to organisms that were not cultured, but were still present on the allograft after decontamination.

A number of studies suggested that the time between asystole and skin preparation could contribute to allograft contamination. However, there were insufficient data to determine whether warm ischemic time or asystole to tissue recovery interval was a better predictor of contamination. One study examined the effects of variable warm ischemic times and was able to determine a precise relationship with cell viability, but the bioburden was not addressed (Niwaya et al. 1995).

Finally, all of the studies did not address the bioburden reduction capabilities of the tissue recovery methods used. As opposed to the contamination rate, the reduction in bioburden value can quantitatively show the effectiveness of decontamination methods, and allows for further optimization.

## Conclusions

Understanding the factors that may promote or inhibit microorganism contamination of allografts during tissue recovery can decrease the number of discarded tissues and/or processed allografts, and could improve the safety of allograft transplantation by minimizing the potential for infection or other complications post surgery. The results of this review suggest that minimizing recovery times (<24 h) and the number of personnel performing tissue recovery are the greatest factors affecting the rate of tissue contamination at or following recovery. Reduction of the contamination rate is also associated with reduced recovery time (i.e., warm ischemic times <6 h), and the use of exclusive recovery sites such as an operating room also result in a marked decrease in the contamination rate. The experience of the recovery team, as well as the number of recovery personnel may also affect the level of contamination observed. The use of povidone iodine to decontaminate skin, multiple sets of sterile instruments, and double gloving do not appear to result in a great reduction of the contamination rate. Due to the lack of information, it cannot be concluded if the use of barriers and/or hygienic precautions taken by personnel to reduce transmission of contaminants have an effect on the contamination rate or bioburden following tissue recovery. Controlled studies in this area are lacking and should be pursued by tissue banking professionals so methods employed to reduce or control bioburden are supported by evidence-based data.

## Electronic supplementary material

Below is the link to the electronic supplementary material.

Supplementary material 1 (PDF 56 kb)

Supplementary material 2 (PDF 82 kb)

Supplementary material 3 (PDF 70 kb)

Supplementary material 4 (PDF 77 kb)

Supplementary material 5 (PDF 211 kb)

Supplementary material 6 (PDF 112 kb)

Supplementary material 7 (PDF 117 kb)
