# Supplementary material for: Tissue recovery practices and bioburden: a systematic review
Source: Cell Tissue Bank. 2016 Oct 19;17(4):561–71. doi: 10.1007/s10561-016-9590-5 (PMC5116036; doi:10.1007/s10561-016-9590-5)
Supplement: Supplementary file 1 — Supplementary material 1 (PDF 56 kb) [file 10561_2016_9590_MOESM1_ESM.pdf]

APPENDIX A: SUMMARY OF SEARCH STRATEGIES

MEDLINE

1 exp Musculoskeletal System/
2 exp "Bone and Bones"/
3 exp Ligaments/
4 exp Tendons/
5 musculoskeletal*.mp.
6 bone?.mp.
7 ligament*.mp.
8 tendon*.mp.
9 (osteoarticular or osteo-articular).mp.
10 soft tissue?.mp.
11 or/1-10
12 Heart Valves/
13 Aortic Valve/
14 Pulmonary Valve/
15 Aorta/
16 Pulmonary Artery/
17 ((heart* or cardiac*) adj2 valve?).mp.
18 (aortic* or aorta*).mp.
19 (pulmonary adj2 valve?).mp.
20 (pulmonary adj2 arter*).mp.
21 or/12-20
22 exp Skin/
23 (skin or derm*).mp.
24 or/22-23
25 11 or 21 or 24
26 Cryopreservation/
27 Tissue Preservation/
28 Tissue Banks/
29 Bone Banks/
30 Refrigeration/
31 Transportation/
32 (cryopreserv* or cryo-preserv*).mp.
33 (tissue? adj2 preserv*).mp.
34 (tissue? adj2 recover*).mp.
35 (tissue? adj2 storag*).mp.
36 (tissue? adj2 transport*).mp.
37 ((tissue? or bone? or skin) adj2 (bank? or banking)).mp.
38 (skin adj2 prep*).mp.

39 refrigeration*.mp.
40 (ischem* adj2 limit*).mp.
41 (body adj2 cooling).mp.
42 asystole*.mp.
43 (zone adj2 recover*).mp.
44 (autopsy adj2 recover*).mp.
45 (organ donat* adj2 recover*).mp.
46 ((personnel? or staff?) adj2 (suit* or proper*)).mp.
47 or/26-46
48 25 and 47
49 exp animals/ not (exp humans/ and exp animals/)
50 48 not 49
51 limit 50 to (case reports or clinical conference or congresses or consensus development conference or consensus development conference, nih)
52 50 not 51
53 limit 52 to (english language and yr="1990 -Current")

EMBASE

1 exp musculoskeletal system/
2 exp bone/
3 exp ligament/
4 exp tendon/
5 soft tissue/
6 musculoskeletal*.mp.
7 bone?.mp.
8 ligament*.mp.
9 tendon*.mp.
10 soft tissue?.mp.
11 (osteoarticular or osteo-articular).mp.
12 or/1-11
13 exp heart valve/
14 Aortic Valve/
15 aorta/
16 pulmonary artery/
17 pulmonary valve/
18 ((heart* or cardiac*) adj2 valve?).mp.
19 (aortic* or aorta*).mp.
20 (pulmonary adj2 valve?).mp.
21 (pulmonary adj2 arter*).mp.
22 or/13-21
23 exp skin/

24 (skin or derm*).mp.

25 or/23-24

26 12 or 22 or 25

27 cryopreservation/

28 tissue preservation/

29 (cryopreserv* or cryo-preserv*).mp.

30 (tissue? adj2 preserv*).mp.

31 (tissue? adj2 recover*).mp.

32 (tissue? adj2 storag*).mp.

33 (tissue? adj2 transport*).mp.

34 ((tissue? or bone? or skin) adj2 (bank? or banking)).mp.

35 (skin adj2 prep*).mp.

36 refrigeration*.mp.

37 (ischem* adj2 limit*).mp.

38 (body adj2 cooling).mp.

39 asystole*.mp.

40 (zone adj2 recover*).mp.

41 (autopsy adj2 recover*).mp.

42 (organ donat* adj2 recover*).mp.

43 ((personnel? or staff?) adj2 (suit* or proper*)).mp.

44 or/27-43

45 26 and 44

46 (exp animals/ or exp animal experimentation/) not ((exp animals/ or exp animal experimentation/) and exp human/)

47 45 not 46

48 limit 47 to (conference abstract or conference paper or conference proceeding or "conference review" or report)

49 47 not 48

50 limit 49 to (english language and yr="1990 -Current")

51 limit 50 to embase
