# Supplementary material for: Tissue recovery practices and bioburden: a systematic review
Source: Cell Tissue Bank. 2016 Oct 19;17(4):561–71. doi: 10.1007/s10561-016-9590-5 (PMC5116036; doi:10.1007/s10561-016-9590-5)
Supplement: Supplementary file 2 — Supplementary material 2 (PDF 82 kb) [file 10561_2016_9590_MOESM2_ESM.pdf]

APPENDIX B: ANALYTICAL FRAMEWORK

Tissue Recovery Time Limits

1. Does ischemic time correlate to bioburden load?
    a. What's the maximum ischemic time from asystole to *Skin Prep* that will result in grafts with the minimum bioburden load*? **Ischemic time** is defined as the time between the cessation of heart beat and the recovery of the tissue. **Skin Prep** is defined as the application of antiseptic solution to decontaminate the skin.*

2. Does *body cooling* or lack thereof correlate to bioburden load? ***Body cooling** is defined as the placement of a donor body in conditions of cold environment.*

3. Does the length of the recovery procedure correlate to bioburden load?
    a. What recovery time range is optimal for controlling bioburden load? *Optimal is defined as the lowest bioburden load found on recovered tissues.*

Donor Preparation and Recovery

4. Do any of these variables correlate to bioburden load?
    a. Donor skin condition ie. cuts or abrasions
    b. Presence of medical interventions
    c. Cleanliness of skin
    d. Trauma
    e. Compound Fractures
    f. Too many variables

5. Do skin preparation parameters correlate to bioburden load?
    a. What skin preparation procedures are optimal for controlling bioburden load? ***Optimal** is defined as the lowest bioburden load found on recovered tissues.* Parameters may include:
        i. Cleaning products
        ii. Cleaning methods
        iii. Shaving
        iv. Disinfection products
        v. Disinfection methods

6. Do physical barriers reduce the risk of tissue contamination?
    a. What are the most effective physical barriers to reduce bioburden transfer to tissues? Variables to compare:
        i. Staff barrier attire
        ii. Double gloving vrs single gloving
        iii. Cut-resistant gloves vrs. Regular gloves
        iv. Donor draping techniques

v. Liquid disinfectants versus treated adhesives such as commercially marketed Optsite™ or Steri-Drape™?

7. Is the order of steps in which a donor is prepped for recovery impact bioburden load?
    a. What is the optimal order of steps in which a donor must be prepped for tissue recovery that produces the least amount of bioburden load to tissues? *Ex. If drapes won't stick after skin prep application is it equally effective to prepare skin after draping?*

8. Do tissue excision techniques impact bioburden load of recovered tissues?
    a. What excision techniques are most effective in reducing opportunities for bioburden contamination for: skin, heart, bone and soft tissue? Variables to compare:
        i. Zone recovery
        ii. Sequencing
        iii. Instrument handling
        iv. Incision site selection
        v. Incision site preparation
        vi. Glove changes between tissues

9. Does post excision tissue handling impact bioburden load?
    a. What are the most effective post excision handling practices that minimize bioburden load? *Handling practices are defined as those from excision of the tissue to storage and transport to the bank.* Variables to compare:
        i. Tissue disinfection treatment prior to packaging
        ii. Packaging materials used

10. Does recovery of tissue after an autopsy or after organ donation affect the bioburden load on recovered tissue?

Tissue Storage and Transport

11. Does the temporary storage conditions impact bioburden load?
    a. What temporary storage method is most effective in controlling bioburden load for each tissue type during transportation? Variables to compare for impact on bioburden:
        i. Storage medium for each tissue (skin, bone, cardiac)
        ii. Optimal temperature for medium
        iii. Optimal storage temperatures for each tissue
        iv. Length of storage time
        v. Temporary packaging
        vi. Shipping container
        vii. Packing configuration

Suitability of Recovery Personnel

12. Can personal hygiene or cleanliness (shower clean?) affect tissue bioburden?

13. Can an acute illness such as exhibiting flu-like symptoms (upper respiratory, lower GI, a fever that causes sweating, or that can affect clear thinking) affect tissue bioburden?

14. Can presence of open lesions (from recovery personnel) affect tissue bioburden?
