# Supplementary material for: Tissue recovery practices and bioburden: a systematic review
Source: Cell Tissue Bank. 2016 Oct 19;17(4):561–71. doi: 10.1007/s10561-016-9590-5 (PMC5116036; doi:10.1007/s10561-016-9590-5)
Supplement: Supplementary file 3 — Supplementary material 3 (PDF 70 kb) [file 10561_2016_9590_MOESM3_ESM.pdf]

APPENDIX C: EXCLUDED STUDIES

Excluded Articles	Reason
Narhi, 2013	Questionnaire results from multiple tissue banks regarding types of pathogens tested and stem cells
Ohkawara, 2012	Does not report any tissue or bioburden outcomes.
Saegman, 2008	Laboratory study on microbial inhibition. No tissue studied.
Vangsness, 2006	Review article
Holder, 1999	Analysis of tissue culture medium and antimicrobial solutions. No tissues studied.
Tuppin, 1997	Census
Myers, 1996	Commentary
Mellonig, 1995	Study of dental tissue
Marx, 1993	Review article

Excluded references

Holder, I. A., Robb, E., & Kagan, R. (1999). Antimicrobial mixtures used to store harvested skin: antimicrobial activities tested at refrigerator (4ºC) temperatures. *Journal of burn care & research*, *20*(6), 501-504.

Marx, R. E., & Carlson, E. R. (1993). Tissue banking safety: caveats and precautions for the oral and maxillofacial surgeon. *Journal of oral and maxillofacial surgery*, *51*(12), 1372-1379.

Mellonig, J. T. (1995). Donor selection, testing, and inactivation of the HIV virus in freeze-dried bone allografts. Practical Periodontics & Aesthetic Dentistry, 7(6), 13-22; quiz 23.

Myers, S. R., Machesney, M. R., Warwick, R. M., & Cussons, P. D. (1996). Skin storage. *British medical journal*, *313*(7055), 439.

Närhi, M., Natri, O., Desbois, I., Kinggaard Holm, D., Galea, G., Aranko, K., ... & Nordstrom, K. (2013). Collection, processing and testing of bone, corneas, umbilical cord blood and haematopoietic stem cells by European Blood Alliance members. *Vox sanguinis*, *105*(4), 346-354.

Ohkawara, H., Kitagawa, T., Fukushima, N., Ito, T., Sawa, Y., & Yoshimine, T. (2012). A Newly Developed Container for Safe, Easy, and Cost-effective Overnight Transportation

of Tissues and Organs by Electrically Keeping Tissue or Organ Temperature at 3 to 6° C. *Transplantation proceedings, 44*(4), 855-858.

Saegeman, V. S., Ectors, N. L., Lismont, D., Verduyckt, B., & Verhaegen, J. (2008). Short-and long-term bacterial inhibiting effect of high concentrations of glycerol used in the preservation of skin allografts. *Burns*, *34*(2), 205-211.

Tuppin, P., Auvert, B., Loty, B., Paulmier, F., & Golmard, J. L. (1997, March). National census of tissue procurement, preservation, and transplantation in France. *Transplantation proceedings, 29*(1), 981-982.

Vangsness, C. T., Wagner, P. P., Moore, T. M., & Roberts, M. R. (2006). Overview of safety issues concerning the preparation and processing of soft-tissue allografts. *Arthroscopy: The Journal of arthroscopic & related surgery*, *22*(12), 1351-1358.
