# Supplementary material for: Tissue recovery practices and bioburden: a systematic review
Source: Cell Tissue Bank. 2016 Oct 19;17(4):561–71. doi: 10.1007/s10561-016-9590-5 (PMC5116036; doi:10.1007/s10561-016-9590-5)
Supplement: Supplementary file 4 — Supplementary material 4 (PDF 77 kb) [file 10561_2016_9590_MOESM4_ESM.pdf]

Appendix D: Microbial Sampling Procedures

First Author, Year	Organisms Tested	Sample Preparations	Microbial Testing Method (Culturing, Serological, Nucleic Acid ID[1])	Media type	Incubation Conditions
Laboratory Studies					
Gaucher, 2012	NR	NR	NR	NR	NR
Ohkawara, 2012	NR	NR	NR	NR	NR
Jashari, 2007	Bacteria, fungi	Tissue fragments, membrane (transport solution flushed through), decontamination solution, cryopreservation solution	Culturing	Thioglycolate (with resazurine) or Sabouraud medium,	37°C for 14 days
				Subcultures for aerobes/anaerobes, trypto-casein & soy medium for fungi & yeasts	20°C-25°C for 14 days
Castagnoli, 2003	NR	NR	NR	NR	NR
Bravo, 2000	NR	NR	NR	NR	NR
Wester, 1998	NR	NR	NR	NR	NR
Armiger, 1995	NR	NR	NR	NR	NR
Niwaya, 1995	NR	NR	NR	NR	NR
Clinical Studies					
Heng, 2013	Bacteria, Fungi, Virus	NR	NR	NR	NR
Schubert, 2012	Bacteria	Tissue Swab	Culturing	Thioglycollate broth, blood agar and chocolate agar	7 days
Gocke, 2005	Bacteria, fungi, Virus	Blood & tissues	Culturing	NR	NR

			Serology for HIV-1 and -2 (Ab), HIV-1 (PCR), hepatitis B virus, hepatitis C virus, human T-lymphocyte virus, syphilis		
Goffin, 2000	Bacteria, Fungi, Virus	"Various tissue samples and working solutions"	NR	NR	NR
Sommerville, 2000	Bacteria, Fungi, Virus	Swab of femoral head, acetabular head, Tissue samples (bone biopsy, capsule)	Culturing	Cultured on blood and chocolate agar, and Brewers Liquid culture medium	7 days
			Serology		
Journeaux, 1999	Bacteria	Swab of bone, bone piece, joint swab, capsule segment	Culturing	Fragments macerated and culture in thioglycollate broth, swabs cultures on chocolate & horse agar & thioglycollate broth	7 days
Verghese, 1999	Bacteria, fungi, viruses	Tissue rinse (multiple) and tissue samples	Culturing	Rinse or samples cultured in thioglycollate broth & RCM, or nutrient broth, or SBD, or SBD with 80μg/ml gentamycin and 200μg/ml chloramphenicol	37°C or room temperature
			Serology for HIV, hepatitis B, hepatitis C, syphilis		

Bettin, 1998	Bacteria, fungi	Samples from bone and soft tissue	Culturing	Dextrose and Schaedler broths. Kimmig, blood agar, chocolate and endo agar plates used.	10 days
Goffin, 1996	Bacteria, fungi, viruses	NR	Serology for HIV-1 and -2 (Ab), HIV-1 (PCR), human T-lymphocyte virus1, hepatitis B, hepatitis C, syphilis, Q fever	NR	NR
Campbell, 1995	Bacteria, virus	Swab of cut bone, synovial fluid	Culturing<br>Serology for HIV-1/2, hepatitis B/C, syphilis	Stuart's transport medium, blood agar, culture broth	7 days
Gall, 1995	Bacteria, fungi	Pericardial swab, tissue, rinse/incubation/freezing solution	Culturing	Blood agar, SDB slopes for swabs. Thioglycolate broth & nutrient broth for tissue samples.	35°C for 48 hours. SBD slopes: 30°C for 21 days
Chapman, 1992	Bacteria	NR	Culturing	Broth, subculture on blood agar	24 h for each culture

[1]Serological analysis includes antibody and antigen using ELISAs or other methods. Nucleic acid Identification can be for RNA or DNA. NR: Not reported
