# Supplementary material for: Tissue recovery practices and bioburden: a systematic review
Source: Cell Tissue Bank. 2016 Oct 19;17(4):561–71. doi: 10.1007/s10561-016-9590-5 (PMC5116036; doi:10.1007/s10561-016-9590-5)
Supplement: Supplementary file 5 — Supplementary material 5 (PDF 211 kb) [file 10561_2016_9590_MOESM5_ESM.pdf]

Appendix E1: Recovery Parameters affecting Bioburden in Laboratory Studies

First Author, Year	Warm Ischemic time	Bioburden reduction methods	Barriers to Bioburden Transmission	Recovery Personnel Precautions	Pre-Recovery skin sanitation	Tissue Recovery Methods	Bioburden following recovery	Isolated Microorganisms
Jashari, 2007	<6 h warm ischemic time	Equipment sterilized	NR	NR	NR	NR	23.75% (19/80) (first step)<br>25.54% (47/184) (modified antibiotic)<br>30.77% (56/182) (classic antibiotic)	*Staphylococci, Citrobacter, Escherichia coli, Pseudomonas, Streptococci, Klebsiella, Propionibacter Bacillus, Moraxella, Enterococci*
Armiger, 1995	<46.5 h between asystole to recovery	NR	NR	NR	NR	NR	NR	NR
Niwaya, 1995	<8.7h (average 3.75 h)	NR	NR	NR	NR	NR	NR	NR

First Author, Year	Warm Ischemic time	Bioburden reduction methods	Barriers to Bioburden Transmission	Recovery Personnel Precautions	Pre-Recovery skin sanitation	Tissue Recovery Methods	Bioburden following recovery	Isolated Microorganisms
Gaucher, 2012	NR	NR	NR	NR	NR	Electric dermatome	NR	NR
Castagnoli, 2003	NR	NR	NR	NR	NR	NR	NR	NR
Bravo, 2000	<15h (warm)<br><24 h (if cooled within 12h)	NR	NR	NR	Standard surgical preparation, standard skin graft procedures	Dermatome	NR	NR
Wester, 1998	NR	NR	NR	NR	NR	Dermatome	NR	NR

NR: Not reported.

Warm ischemic time: Time period between asystole and skin preparation while warm (room temperature).

Appendix E2: Recovery Parameters Affecting Bioburden for Clinical Studies

First Author, Year	Warm Ischemic time	Bioburden reduction methods	Barriers to Bioburden Trans-mission	Recovery Personnel Precautions	Pre-Recovery Skin Sanitation	Tissue Recovery Methods	Bioburden following recovery	Isolated Microorganisms
Heng, 2013	<15h (warm)<br><24 h (if cooled within 12h)	Recovery site exclusive for tissue harvesting	Care not to enter the donor's trachea or abdominal cavity during excisions	NR	NR	Heart valve block excised	13.9% (5/36) (penicillin/streptomycin)<br>42.9% (9/21) (amikacin/vancomycin)	*Propionibacterium acnes, Pseudomonas, Staphylococcus, Escherichia colo, Acinetobacter, Micrococcus, Staphylococcus aureus, MRSA, Rhodococcus, Alpha haemolytic streptococcus, bacillus, Candida albicans, Candida parapsilosis*
Goffin, 2000	<6h warm ischemic time	NR	NR	NR	"sterile conditions" or "clean conditions"	NR	25.4% (441/1739)<br>353/441 Multi-organ or	NR

First Author, Year	Warm Ischemic time	Bioburden reduction methods	Barriers to Bioburden Trans-mission	Recovery Personnel Precautions	Pre-Recovery Skin Sanitation	Tissue Recovery Methods	Bioburden following recovery	Isolated Microorganisms
							transplant<br><br>86/441 cadavers	
Verghese, 1999	<24h	Sterile instruments used from pericardium onwards	Sterile gowns, gloves	NR	NR	NR	NR	Gram negative: *Klebsiella, Enterobacter, E. coli, Citrobacter, Proteus, Pseudomonas, Flavobacterium, Acinetobacter, Alkaliigenes*<br><br>Gram positive: *Enterococcus, diphtheroids, Micrococci, Staphylococcus, Streptococci, Clostridium, Mycobacteria,*

First Author, Year	Warm Ischemic time	Bioburden reduction methods	Barriers to Bioburden Trans-mission	Recovery Personnel Precautions	Pre-Recovery Skin Sanitation	Tissue Recovery Methods	Bioburden following recovery	Isolated Microorganisms
								Streptomyces<br>Fungi: *Candida krusei, Candida tropicalis, Candida parapsilosis, Candidaguillemondii, Gotrichum,*<br>*Aspergillus sp, Aspergillus fumgatus, Aspergilllus terreus*<br>*Aspergillus niger, Aspergillus flavus Penicillium, Paeceliomyces sp. Curvularia, Trichoderma, Exophilia*
Goffin,	<6h warm	NR	NR	NR	NR	Cold sterile	15.9%	Gram positive

First Author, Year	Warm Ischemic time	Bioburden reduction methods	Barriers to Bioburden Trans-mission	Recovery Personnel Precautions	Pre-Recovery Skin Sanitation	Tissue Recovery Methods	Bioburden following recovery	Isolated Microorganisms
1996	ischemic time					transport media, including saline, Ringer's solution, Eurocollins, Tissue Culture Medium<br><br>199 HEPES (TCM 199)	positive culture (155/974)	cocci & rods, Gram negative, anaerobic bacteria, yeast& fungi
Gall, 1995	<24h (average 15 h) from asystole to recovery	NR	NR	Sterile draping	TPCH Surgeons (short OR exposure)	Surgical standard skin sterilization	2% (1/47) heart transplant<br><br>14% (10/71) Multi-organ (without antibiotics)	*Staphylococcus epidermidis, Candida albicans, S. aureus, Acinetobacter, Diphtheroid, viridans. Streptococcus*,

First Author, Year	Warm Ischemic time	Bioburden reduction methods	Barriers to Bioburden Trans-mission	Recovery Personnel Precautions	Pre-Recovery Skin Sanitation	Tissue Recovery Methods	Bioburden following recovery	Isolated Microorganisms
							9% (5/53) Multi-organ (with antibiotics)<br>54% (184/345) mortuary	hemolytic S*treptococcus*, *pseudomonas,* Gram negative *Bacillus*
Schubert, 2012	<24 h (15 cases)	NR	NR	Team of 2-6 surgeons, varying experience	NR	NR	4.7% (116/2447 bone samples)	Coagulase neg. *Staphylococcus, Micrococcus, Bacillus cereus, Corynebacterium, Penicilium, Alcaligenes, Lactobacillus, Escherichia coli, Acinetobacter, Enterococcus, Pseudomonas aeruginosa, Pneumococcus,*

First Author, Year	Warm Ischemic time	Bioburden reduction methods	Barriers to Bioburden Trans-mission	Recovery Personnel Precautions	Pre-Recovery Skin Sanitation	Tissue Recovery Methods	Bioburden following recovery	Isolated Microorganisms
								Neisseria, Candida
Gocke, 2005	NR	NR	NR	Donor assessment done by specialty nurses, recovery done by trained surgical technicians	NR	NR	45% positive serology	HIV, Hepatitis B, Hepatitis C, syphilis, HTLV
Sommerville, 2000	NR	Prophylactic antibiotic	NR	NR	Standard surgical procedures	NR	22% (51/232)	*Staphylococcus epidermidis, Staphylococcus Corynebacterium, Proprionibacterium, Bacillus,* Diphtheroids, N*eisseria, Serratia marcescens,*

First Author, Year	Warm Ischemic time	Bioburden reduction methods	Barriers to Bioburden Trans-mission	Recovery Personnel Precautions	Pre-Recovery Skin Sanitation	Tissue Recovery Methods	Bioburden following recovery	Isolated Microorganisms
								Pseudomonas, Acinetobacter Enterobacter Aspergillus, Candida, Streptomyces
Journeaux, 1999	NR	NR	NR	NR	NR	NR	%13 (30/232 Live)<br>24% of Multi-organ samples (65/272)<br>35% of cadaveric samples (53/151)	Coagulase negative *Staphylococcus, Viridans* and non-hemolytic *Streptococci, Enterobacteria,*<br>*Corynebacterium, Propionibacterium*

First Author, Year	Warm Ischemic time	Bioburden reduction methods	Barriers to Bioburden Trans-mission	Recovery Personnel Precautions	Pre-Recovery Skin Sanitation	Tissue Recovery Methods	Bioburden following recovery	Isolated Microorganisms
Bettin, 1998	<24 hours warm ischemic time<br>(Operating room average 2.5 h; Morgue average 11 h)	Two sets of instruments used separately on each side	Double gloves	NR	10% povidone iodine for 15 min, standard shaving, scrubbing, wrapping procedure	Steridrapes	49% overall (211/431)<br>40% of Cadaveric samples (45/112)<br>51% of organ donors (164/319)	*Staphylococci, Propionibacteria;*<br>*Corynbacteria;*<br>*Sarcina* E*nterococcus,*<br>*E. coli, Candida, Bacteroides, Acinetobacter, Pneumococcus, Pseudomonas*<br>*Peptococcus*<br>Coagulase-negative *Staphylococcus*
Campbell, 1995	NR	Antibiotics during hip surgery	NR	NR	NR	NR	NR	*Staphylococcus epidermidis, Staphylococcus aureus, Streptococci,*

First Author, Year	Warm Ischemic time	Bioburden reduction methods	Barriers to Bioburden Trans-mission	Recovery Personnel Precautions	Pre-Recovery Skin Sanitation	Tissue Recovery Methods	Bioburden following recovery	Isolated Microorganisms
								anaerobic diphtheroids, Gram positive and negative *Bacillus*
Marx, 1993	<24 h If body is refrigerated<br><br><12hrs if body is not refrigerated	Required: air filtration, stainless steel furniture, washable walls. Germicidal solution left on skin and sprayed on incision	NR	NR	Standard surgical' procedure, germicidal solution sprayed on incision	Draped in standard manner	NR	NR
Chapman, 1992	NR	"Antibiotic cover"	NR	NR	NR	NR	22.8% (8/35)<br>18.5% (5/27) femoral	*Streptococcus* group F, *Haemophilus influenzae*, *Staphylococcus*

First Author, Year	Warm Ischemic time	Bioburden reduction methods	Barriers to Bioburden Trans-mission	Recovery Personnel Precautions	Pre-Recovery Skin Sanitation	Tissue Recovery Methods	Bioburden following recovery	Isolated Microorganisms
							heads from living donors	*aureus*, Gram positive diphtheroids, *Staphylococci epidermidis*
							37.5% (3/8) bone allografts from cadaveric donors	
Schubert, 2012	<24 h (15 cases)	NR	NR	NR	Team of 2-6 surgeons, varying experience	NR	2.7% (90/3315 tendon grafts)	Coagulase neg. *Staphylococcus, Micrococcus, Bacillus cereus, Corynebacterium, Penicilium, Alcaligenes, Lactobacillus, Eschericcia coli, Acinetobacter, Enterococcus, Pseudomonas*

First Author, Year	Warm Ischemic time	Bioburden reduction methods	Barriers to Bioburden Trans-mission	Recovery Personnel Precautions	Pre-Recovery Skin Sanitation	Tissue Recovery Methods	Bioburden following recovery	Isolated Microorganisms
								aeruginosa, Pneumococcus, Neisseria, Candida
Gocke, 2005	NR	NR	NR	Donor assessment done by specialty nurses, recovery done by trained surgical technicians	NR	NR	NR	HIV, Hepatitis B/C, syphilis, HTLV, bacteria/fungi NR

NR: Not reported.
Warm ischemic time: Time period between asystole and cold rinse or transport solution
