# Supplementary material for: Tissue recovery practices and bioburden: a systematic review
Source: Cell Tissue Bank. 2016 Oct 19;17(4):561–71. doi: 10.1007/s10561-016-9590-5 (PMC5116036; doi:10.1007/s10561-016-9590-5)
Supplement: Supplementary file 6 — Supplementary material 6 (PDF 112 kb) [file 10561_2016_9590_MOESM6_ESM.pdf]

Appendix F: Recovered Tissue Treatment and Final Outcomes for Laboratory Studies

First Author, Year	Tissue Recovered	Number of Samples Recovered	Temporary storage solution	Long Term Storage Solution and Parameters	Tissue Viability	Proportion of positive cultures or microbial load
Jashari, 2007	Heart, arteries	446	Isotonic solution (saline, Ringers, M199) on ice	Cryopreservation solution	Morphological examination	9.78% (18/184)
						9.89% (18/182)
Armiger, 1995	Aortic, pulmonary valves	94	NR	10% DMSO in M199, <-135°C	Tissue culture, autoradiography	NR
Niwaya, 1995	Heart, pulmonary valves	12	TC-199 culture medium with 10% fetal calf serum (FCS), 5% HEPES buffer	10% DMSO in TC-199 culture medium with 10% fetal calf serum, 5% HEPES, -80°C for 30 days	Flow cytometry, fibroblast viability testing	NR
Gaucher, 2012	Skin	38	Ringer Lactate with antibiotic solution	10% DMSO, 0.7% human serum albumin	MTT assay, histology	NR
			RPMI 1640 with	15% Glycerol, 0.7%		

First Author, Year	Tissue Recovered	Number of Samples Recovered	Temporary storage solution	Long Term Storage Solution and Parameters	Tissue Viability	Proportion of positive cultures or microbial load
			antibiotic solution	human albumin		
Castagnoli, 2003	Skin	350	RPMI 1640, 1% human serum albumin with antibiotics	TM medium<br>4 or 37°C, up to 1 month<br>sterile containers<br>10% DMSO, <-135°C, 15-45 days. Polystyrene boxes in aluminum heat sink boxes	MTT assay<br>25% decrease in viability after 12 to 30 h post-procurement (fresh). Decreased viability by 50% by 60 h post-procurement. Skin stored at 4°C showed slower decreases in viability compared to 37°C. Cryopreserved skin has comparable viability of fresh skin stored at 4	NR

First Author, Year	Tissue Recovered	Number of Samples Recovered	Temporary storage solution	Long Term Storage Solution and Parameters	Tissue Viability	Proportion of positive cultures or microbial load
					◦C for 4 days.	
Bravo, 2000	Skin	46 (cadaveric)	Saline Rinse	10% DMSO (1.4M)	Tetrazolium reduction assay, oxygen consumption rate	NR
		14 (living)	Saline saturated sterile gauze	10% glycerol (1.37M), -115°C to -196°C, 15-30 days		
Wester, 1998	Skin	NR	MEM with BSS	MEM-BSS with gentamicin at 4°C < 13 days	Anaerobic metabolism	NR

First Author, Year	Tissue Recovered	Number of Samples Recovered	Temporary storage solution	Long Term Storage Solution and Parameters	Tissue Viability	Proportion of positive cultures or microbial load
				MEM-BSS with gentamicin<br>-22°C for >24 h		

NR: Not reported
