# Supplementary material for: Tissue recovery practices and bioburden: a systematic review
Source: Cell Tissue Bank. 2016 Oct 19;17(4):561–71. doi: 10.1007/s10561-016-9590-5 (PMC5116036; doi:10.1007/s10561-016-9590-5)
Supplement: Supplementary file 7 — Supplementary material 7 (PDF 117 kb) [file 10561_2016_9590_MOESM7_ESM.pdf]

**Appendix G: Recovered Tissue Treatment and Final Outcomes for Clinical Studies**

| First Author, Year | Tissue Recovered | Number of Samples Recovered | Temporary storage solution | Long Term Storage Solution and Parameters | Tissue Viability | Proportion of Positive Cultures or Microbial Load Post-Processing | Shipping Solution and Parameters |
|---|---|---|---|---|---|---|---|
| Heng, 2013 | Aortic, pulmonary mitral valves | 57 | 1-10°C saline | 10% DMSO in M199<br>-196°C for up to 5 years.<br>Double cryogenic bags. | Valves visually inspected, tested for leakage/regurgitation with saline (prior to disinfection). Atrial and ventricular myocardium, aorta and pulmonary artery examined histopathologically. | 11.1% (4/36)<br>4.76% (1/21) | Transported in charged dry shipper<br>at<br>-135°C |
| Goffin, 2000 | Cardiac allografts (heart & arterial roots) | 1817 hearts, 12 semilunar valves | cold-ice sterile medium (ringer, saline, eurocollins, M199) | 10% DMSO, -140°C to -190°C | Leaflet competence test | 5.8% (100/1739) | 10% DMSO, dry shipper,<br>-140°C to -190°C<br>* |

| First Author, Year | Tissue Recovered | Number of Samples Recovered | Temporary storage solution | Long Term Storage Solution and Parameters | Tissue Viability | Proportion of Positive Cultures or Microbial Load Post-Processing | Shipping Solution and Parameters |
|---|---|---|---|---|---|---|---|
| | | | | | | | |
| Verghese, 1999 | Heart (whole) | 302 | HBSS (salt solution) with antibiotics, on ice | 10% DMSO in M199, -120°C to -170°C | NR | NR | Samples placed into sterile plastic bags and immersed in Hank's balanced salt solution with antibiotics. It is stored in an ice pack surrounded by sterile ice for transportation. |
| Goffin, 1996 | Cardiac allografts (heart & arterial roots) | 989 | cold-ice sterile medium (Ringer, saline, Eurocollins, M199) | 10% DMSO in TCM 199, -150°C, double bag | Leaflet leakage tested | 4.1% (43/974) | Dry shipper or air, 150°C or dry ice |

| First Author, Year | Tissue Recovered | Number of Samples Recovered | Temporary storage solution | Long Term Storage Solution and Parameters | Tissue Viability | Proportion of Positive Cultures or Microbial Load Post-Processing | Shipping Solution and Parameters |
|---|---|---|---|---|---|---|---|
| Gall, 1995 | Cardiac valves (whole heart, heart valve block) | 680 | M199 with penicillin/streptomycin (Cadaver/ multi organ)<br><br>No solution (Multi-organ/donor) | 10% DMSO in M199, -135°C | NR | 0% ( 0/47) Heart transplant<br>21% (21/99) Multi-organ (no abx)<br>2% (1/61) Multi-organ (with abx)<br>3% (9/345) (mortuary) | Wet ice (whole heart), or cold packs (valve blocks) |
| Schubert, 2012 | Bone | 2447 | Sterile dry container at 4°C | NR | NR | 18.8% (39/208; no antibiotics)<br>8% (42/527; 0.1% thimerosal)<br>4.6% (79/1712; 1.2g/l rifampicin) | NR |
| Gocke, 2005 | Bone and tissue | 297,644 samples<br>4488 | NR, shipped on ice overnight | NR | NR | NR | On ice, overnight |

| First Author, Year | Tissue Recovered | Number of Samples Recovered | Temporary storage solution | Long Term Storage Solution and Parameters | Tissue Viability | Proportion of Positive Cultures or Microbial Load Post-Processing | Shipping Solution and Parameters |
|---|---|---|---|---|---|---|---|
| | | donors | | | | | |
| Sommerville, 2000 | Bone (femoral head) | 232 | None | NR | Not Done | NR | NR |
| Journeaux, 1999 | Bone (femur, tibia, hemipelvis) | 401 | NR | NR | NR | NR | NR |
| Bettin, 1998 | Bone | 431 | NR | NR | NR | NR | NR |
| Campbell, 1995 | Bone (femoral head/long bones) | 2361 | Wrapped in sterile bags | -70°C | Histopathology | 1.1% (27/2361) | NR |
| Chapman, 1992 | Bone (femoral head/whole bones) | 35 | NR | NR | NR | 0% (0/30) (5 discarded) | NR |
| Schubert, 2012 | Tendon | 3315 | Sterile dry container at | NR | NR | 6.9% (7/101; no antiseptics) | NR |

| **First Author, Year** | **Tissue Recovered** | **Number of Samples Recovered** | **Temporary storage solution** | **Long Term Storage Solution and Parameters** | **Tissue Viability** | **Proportion of Positive Cultures or Microbial Load Post-Processing** | **Shipping Solution and Parameters** |
|---|---|---|---|---|---|---|---|
| | | | 4°C | | | 10.4% (12/115; 90% alcohol)<br>4.6% of (143/3099; 0.02% chlorohexadine in 70% alcohol) | |
| Gocke, 2005 | Tendon, fascia | NR | NR | NR | NR | NR | NR |

NR: Not reported
